# Lipodystrophy in Central Asia: A Regional Literature Review with a Case Series of Congenital and Acquired Generalized Lipodystrophy

**DOI:** 10.3390/metabo16090678

**Published:** 2026-09-14

**Authors:** Marzhan Rakhimzhanova, Bikadisha Bimurat, Aimira Jangashkarova, Assel Issabayeva, Aigul Durmanova, Alina Alzhaxina, Tatyana Ivanova-Razumova, Gulmira Rapilbekova

**Affiliations:** 1 Department of Pediatrics, Corporate Fund “University Medical Center”, Astana 010000, Kazakhstan; bikadisha.bimurat@nu.edu.kz (B.B.); aimiraa.dzh@gmail.com (A.J.); t.ivanova.razumova@umc.org.kz (T.I.-R.); gulmira.rapilbekova@umc.org.kz (G.R.); 2 Department of Biomedical Sciences, School of Medicine, Nazarbayev University, Astana 010000, Kazakhstan; doc.issabayeva@gmail.com; 3 Department of Internal Medicine, Corporate Fund “University Medical Center”, Astana 010000, Kazakhstan; aigul.durmanova@umc.org.kz; 4 Department of Complex Somatics, Multidisciplinary Children’s City Hospital, Astana 010000, Kazakhstan; alina970604@gmail.com

**Keywords:** lipodystrophy, congenital generalized lipodystrophy, acquired generalized lipodystrophy, Central Asia, rare diseases, insulin resistance, genetic testing, healthcare disparities

## Abstract

*Background*: Lipodystrophy syndromes are a group of rare disorders characterized by partial or generalized loss of adipose tissue, severe insulin resistance, and multiple metabolic complications. Despite increasing global awareness, lipodystrophy remains underrepresented in the published literature from Central Asia, and its true regional prevalence remains unknown. This study presents a case series of patients from Central Asia with congenital and acquired generalized lipodystrophy, reviews the existing regional literature, and determines challenges and future directions to improve lipodystrophy care in this region. *Methods*: We conducted a targeted regional literature review focusing on published reports from five Central Asian countries: Kazakhstan, Kyrgyzstan, Tajikistan, Turkmenistan, and Uzbekistan. A literature search was performed in electronic databases, including PubMed/MEDLINE, Scopus, and Web of Science, from the earliest available records to 20 July 2026. *Results*: Reported cases of lipodystrophy from Central Asia were extremely scarce. Our cases also highlighted important barriers to effective care, including limited disease awareness and access to specialized therapies. *Discussion*: These findings indicate that lipodystrophy is underrepresented in the literature, and the true disease burden and extent of underdiagnosis in Central Asia remain unknown. Improving clinical awareness, developing international collaborations, widening access to genetic testing, and establishing multidisciplinary referral systems could significantly enhance patient outcomes. *Conclusions*: Lipodystrophy remains an underreported, rare condition in Central Asia. Our findings emphasize the urgent need for improved awareness, regional cooperation, development of expert clinics, and better access to specialized diagnostic and therapeutic resources to optimize care for affected patients.

## 1. Introduction

Lipodystrophy syndromes are a group of rare disorders characterized by total or partial absence of adipose tissue that can give rise to severe metabolic complications and shortened life expectancy [1]. They are classified into four main categories based on the etiology and distribution of fat loss: congenital generalized lipodystrophy (CGL or Berardinelli–Seip syndrome), acquired generalized lipodystrophy (AGL or Lawrence syndrome), familial partial lipodystrophy (FPL), and acquired partial lipodystrophy (APL or Barraquer–Simons syndrome) [2,3]. Among these, CGL typically develops from birth or early infancy, whereas AGL usually manifests later in childhood or adulthood; both conditions may follow a severe clinical course with significant metabolic disturbances.

The overall worldwide prevalence of lipodystrophy is roughly 1.3–4.7 cases per million individuals [4]; the prevalence of generalized lipodystrophy is estimated at about 0.23 cases per million, while partial lipodystrophy is more common, with an estimated 2.84 cases per million [4]. Despite these estimates, the true prevalence of lipodystrophy is likely underestimated due to its phenotypic diversity, frequent misdiagnosis as severe insulin resistance or atypical diabetes, and limited access to genetic testing [5].

Generalized lipodystrophies result from an impairment of adipose tissue development, maintenance, or survival, leading to almost complete loss of functional adipose tissue stores [6,7,8]. In CGL, pathogenic variants in genes such as *AGPAT2*, *BSCL2*, *CAV1*, and *CAVIN1/PTRF* cause failure in adipogenesis, triglyceride synthesis, lipid droplet formation, or caveolae-related adipocyte function, leading to severe deficiency of adipose tissue from birth or early infancy [9]. On the contrary, in AGL, fat tissue distribution is normal at birth and gradually lost later during childhood or adolescence [10,11]. The pathogenesis of AGL is not completely understood and has been proposed to arise from immune-mediated fat tissue destruction, commonly seen together with autoimmune diseases, panniculitis, or dysregulation of the complement system [12,13,14,15]. In both forms, the disruption of fat tissue homeostasis results in ectopic fat deposition in hepatic and skeletal muscle tissues, severe insulin resistance, hypertriglyceridemia, hypoleptinemia, and hepatic steatosis. The common clinical features are muscular appearance; almost total deficiency of fat tissue in the face, limbs, and trunk; dermatologic manifestations (acanthosis nigricans, eruptive xanthomas); a prominent vascular pattern; and polyendocrine metabolic ovarian syndrome (PMOS) [16,17,18,19]. 

Management of generalized lipodystrophies focuses on preventing and controlling metabolic and cardiovascular complications, beginning with lifestyle measures, such as calorie restriction and regular physical activity. The conventional pharmacotherapy involves glucose- and lipid-lowering and antihypertensive drugs. Metreleptin, a recombinant human leptin analog, is the only approved targeted treatment against the metabolic complications of lipodystrophy [20,21,22]; however, its use in Central Asia has not been reported.

Although the global awareness of lipodystrophy has markedly improved over recent years, considerable geographic differences persist. Central Asia remains largely unrecognized in the literature, with very few published reports despite a population exceeding 80 million people. 

This study presents a case series of patients from Central Asia with generalized lipodystrophy, conducts a regional literature review, addresses healthcare challenges, and proposes strategies to improve lipodystrophy care in the region.

## 2. Methods

The study comprised two components: a case series and a regional literature review. The former included two patients with generalized lipodystrophy who were evaluated at the Corporate Fund “University Medical Center.” A targeted regional literature review was performed, rather than a systematic or scoping review, to identify and characterize published evidence on lipodystrophy from five Central Asian countries: Kazakhstan, Kyrgyzstan, Tajikistan, Turkmenistan, and Uzbekistan. The literature search was performed in electronic databases, including PubMed/MEDLINE, Scopus, and Web of Science, from the earliest available records to 20 July 2026. 

The search strategy combined terms related to lipodystrophy with terms referring to Central Asian countries. The keywords used were as follows: “lipodystrophy” or “congenital generalized lipodystrophy” or “acquired generalized lipodystrophy” or “Berardinelli-Seip syndrome” or “Lawrence syndrome” and “Central Asia” or “Central Asian” or “Kazakhstan” or “Kyrgyzstan” or “Tajikistan” or “Turkmenistan” or “Uzbekistan.” Original clinical studies, case reports, case series, and observational studies describing patients with lipodystrophy from any of the five Central Asian countries were considered eligible. Studies on HIV-associated lipodystrophy or isolated injection-related lipoatrophy, animal or experimental studies, and publications without relevant clinical data from Central Asia were excluded. No language restrictions were enforced. Titles and abstracts were screened for relevance, followed by full-text assessment of potentially eligible publications. 

## 3. Clinical Case Presentation

### 3.1. Clinical Case 1

A 1.5-year-old patient was admitted for evaluation. He was born full-term following an uncomplicated third pregnancy and second delivery, with a birth weight of 3410 g and a length of 52 cm, and was the child of healthy, non-consanguineous parents living in the western region of Kazakhstan.

The condition manifested from birth. On days 3–4 after discharge from the maternity ward, the patient developed intermittent fever varying between 37 °C and 40 °C and frequent loose stools. The patient’s mother noted that the newborn had relatively dark skin. At 4 months, the patient was admitted to a local hospital with concerns about excessive linear growth, low body weight, generalized absence of subcutaneous fat, hyperphagia, sweating, skin hyperpigmentation, irritability, and abdominal distention. The patient was diagnosed with suspected secondary malabsorption syndrome, enzymopathy, grade 1–2 hypotrophic malnutrition, perinatal central nervous system injury, and possible celiac disease. Generalized lipodystrophy syndrome was subsequently suspected at 5 months. At the age of 5, the patient was diagnosed with delayed speech development.

On physical examination, the generalized absence of subcutaneous fat tissue and a prominent muscular appearance in the arms and legs were found. Relative to his age, he was tall (height SDS was +2.98) and had excess weight (BMI SDS was +2.59). Bone and craniofacial anomalies included an acromegaloid facial appearance, frontal bossing, enlarged hands and feet, and pectus excavatum. Hypertrichosis was noted on the back and upper extremities; acanthosis nigricans was present in the neck, axillae, and elbow creases; and sparse hair growth was identified on the neck and forehead. The abdomen was distended, the liver was palpable 6 cm below the right costal margin, and the spleen was palpable 4 cm below the left costal margin. Cardiac auscultation revealed muffled heart sounds and a systolic murmur at the apex. The apical impulse was diffuse and sustained.

#### 3.1.1. Molecular Genetic Analysis

Genomic DNA was extracted in 2016 and analyzed at UT Southwestern Medical Center, USA, using whole-exome sequencing (WES). The analysis demonstrated a homozygous variant, c.823C>T (p.R275*), in exon 8 of the *BSCL2* gene, confirming Berardinelli–Seip syndrome (OMIM: 269700). This variant is classified as pathogenic with strong evidence as determined by Franklin Genoox platform (Dallas, TX, USA) and VarSome ACMG Implementation according to the American College of Medical Genetics and Genomics and the Association for Molecular Pathology (ACMG/AMP) criteria. A clinical diagnosis of CGL type 2 was confirmed at 1.5 years based on the molecular genetic analysis. 

#### 3.1.2. Management and Outcome Characteristics

Initially, the treatment strategy included a calorie-restricted diet providing approximately 1100–1200 kcal/day and approximately 20–25 g of protein/day to support age-appropriate growth and development, with fat restriction and limitation of added sugars. The patient’s mother demonstrated high adherence to the prescribed dietary recommendations. After the start of therapy, a decrease in hyperphagia was noted, which facilitated further dietary adherence and was accompanied by a more pronounced improvement in carbohydrate and lipid metabolism.

When the patient was 2.7 years old, he had progressive metabolic abnormalities. During this period, secondary cardiomyopathy and a bicuspid aortic valve were diagnosed. Enalapril 0.8 mg per dose, administered twice daily, and ursodeoxycholic acid 250 mg once daily were recommended. Despite dietary modifications, at the age of 4 years, metabolic dysfunction-associated steatotic liver disease (MASLD) and impaired fasting glucose (IFG) were diagnosed. Metformin 500 mg once daily was prescribed and the dose of enalapril increased to 2.5 mg once daily. At 5.9 years, type 2 diabetes, secondary hypertension, and secondary nephropathy were diagnosed; consequently, the treatment plan was changed to ursodeoxycholic acid 250 mg twice daily, metformin 500 mg twice daily, and enalapril 5 mg twice daily to prevent further progression of cardiometabolic complications.

At the age of 6.5 years, metreleptin was initiated with a dosage of 0.06 mg/kg/day (1.8 mg/day—0.38 mL) administered subcutaneously. Within 10 days, hyperphagia, a key contributor to increased appetite, revealed a significant reduction. The patient’s glycemic control and lipid profile (specifically, triglycerides) improved considerably within six months; given these improvements, metformin was discontinued. 

Seven months after the start of the treatment, an anticipated interruption in metreleptin availability resulted in the dose being gradually reduced from 1.8 mg/day to 0.18 mg/day based on the remaining drug supply, with the aim of avoiding abrupt treatment discontinuation and the potential rebound worsening of hypertriglyceridemia. Due to financial constraints and drug accessibility issues, metreleptin was discontinued twice—after one year and after two years of treatment. During these interruptions (approximately 3 months each), metformin was restarted to manage hyperglycemia as plasma glucose levels began to rise, along with increased fatigue and challenges with control of hunger, which reveals the necessity of metreleptin for maintaining metabolic control.

**Figure 1:**
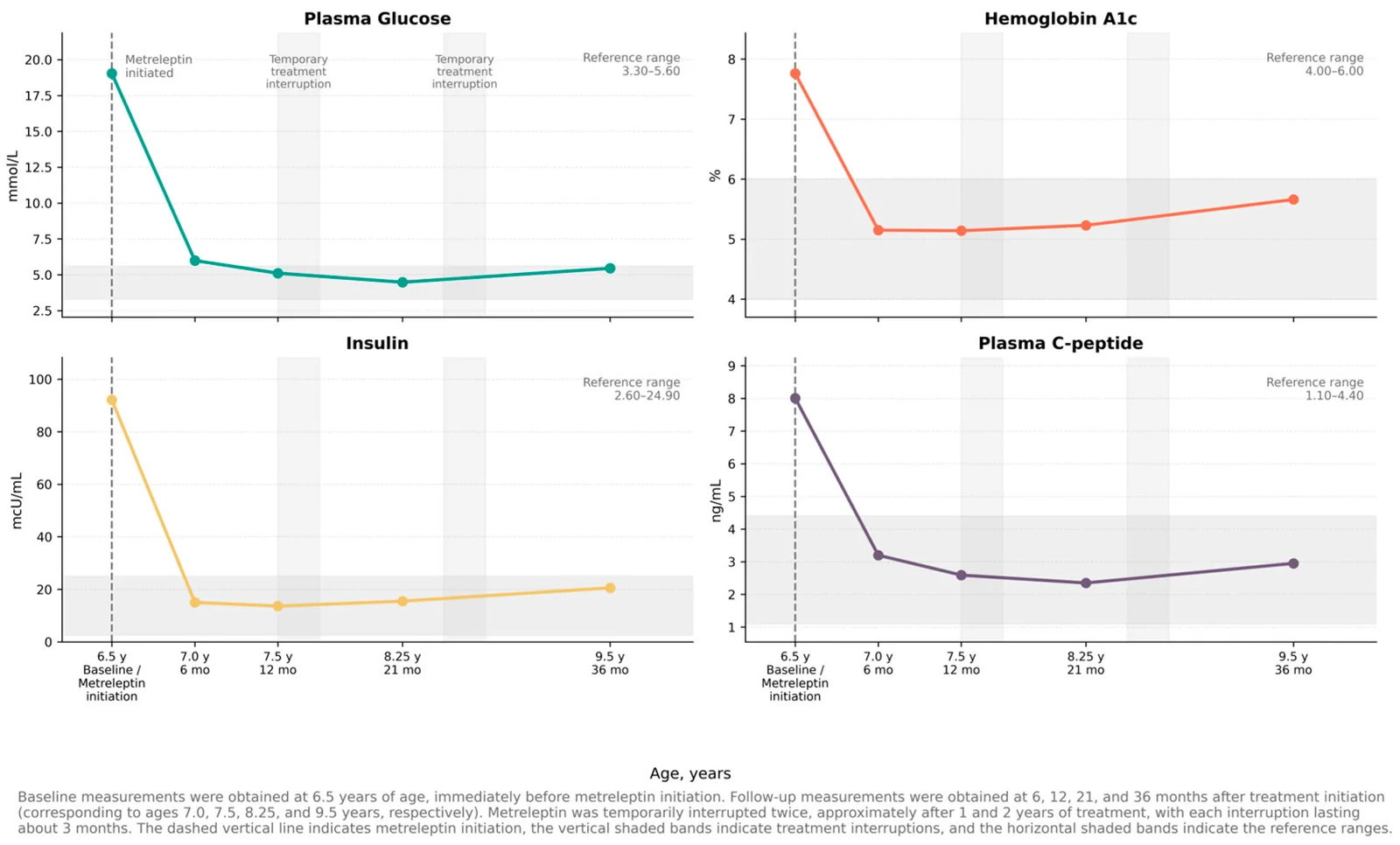
Metabolic response before and during metreleptin therapy.

**Figure 2:**
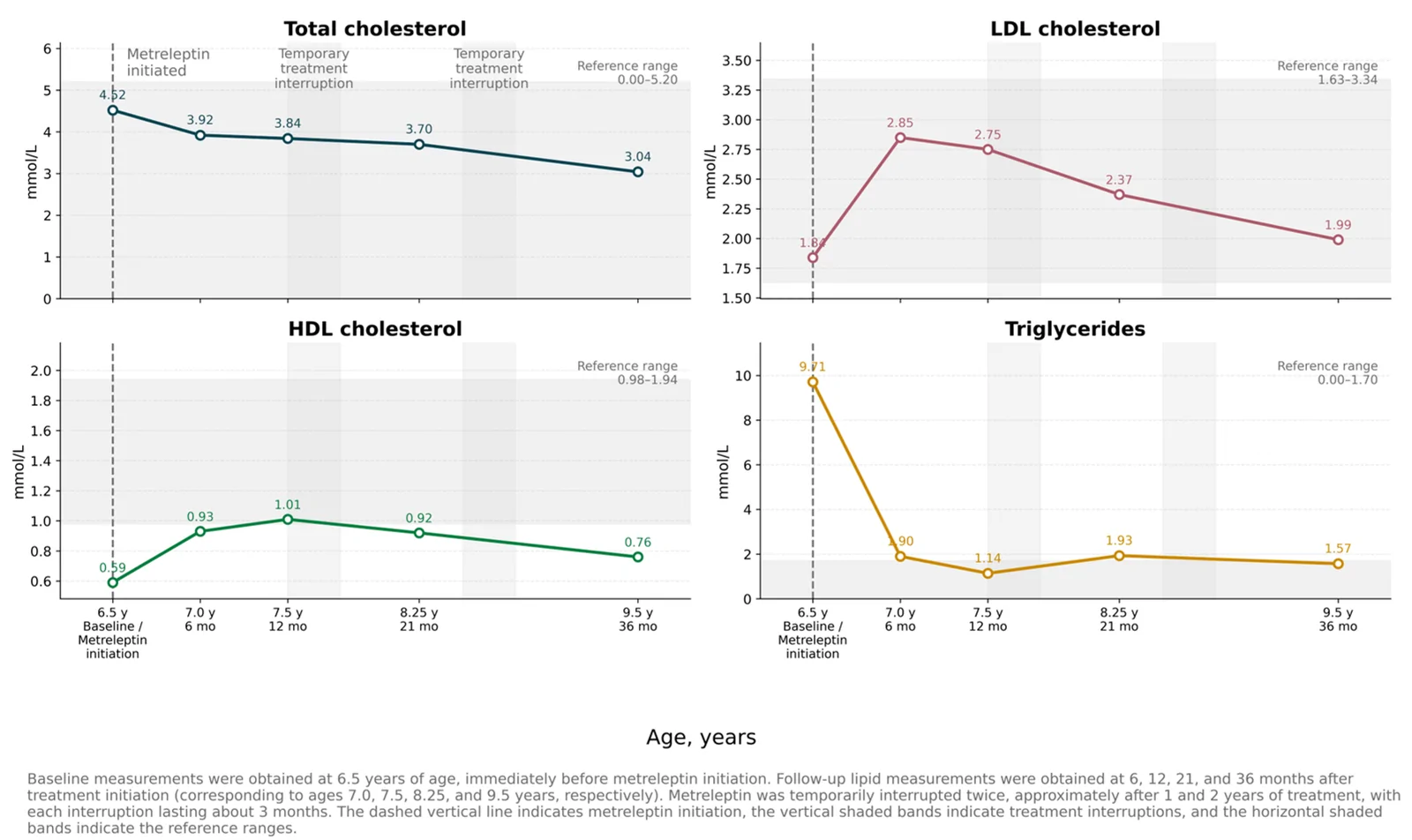
Changes in lipid profile before and during metreleptin therapy.

Despite treatment discontinuations, the patient achieved signiﬁcant metabolic improvements during the 36-month follow-up. Triglyceride levels decreased from 9.71 mmol/L to 1.57 mmol/L, and plasma glucose level markedly reduced from 19.40 mmol/L to 4.48 mmol/L. Moreover, hepatosplenomegaly and nephromegaly were resolved within 6–12 months, and no proteinuria was detected after 6–12 months; blood pressure stabilized without any changes in antihypertensive medication regime; and cardiac function improved, with an ejection fraction of 62%. Changes in glycemic and lipid parameters before and during treatment are presented in Table 1 and Figure 1, and Table 2 and Figure 2, respectively.

**Figure 3:**
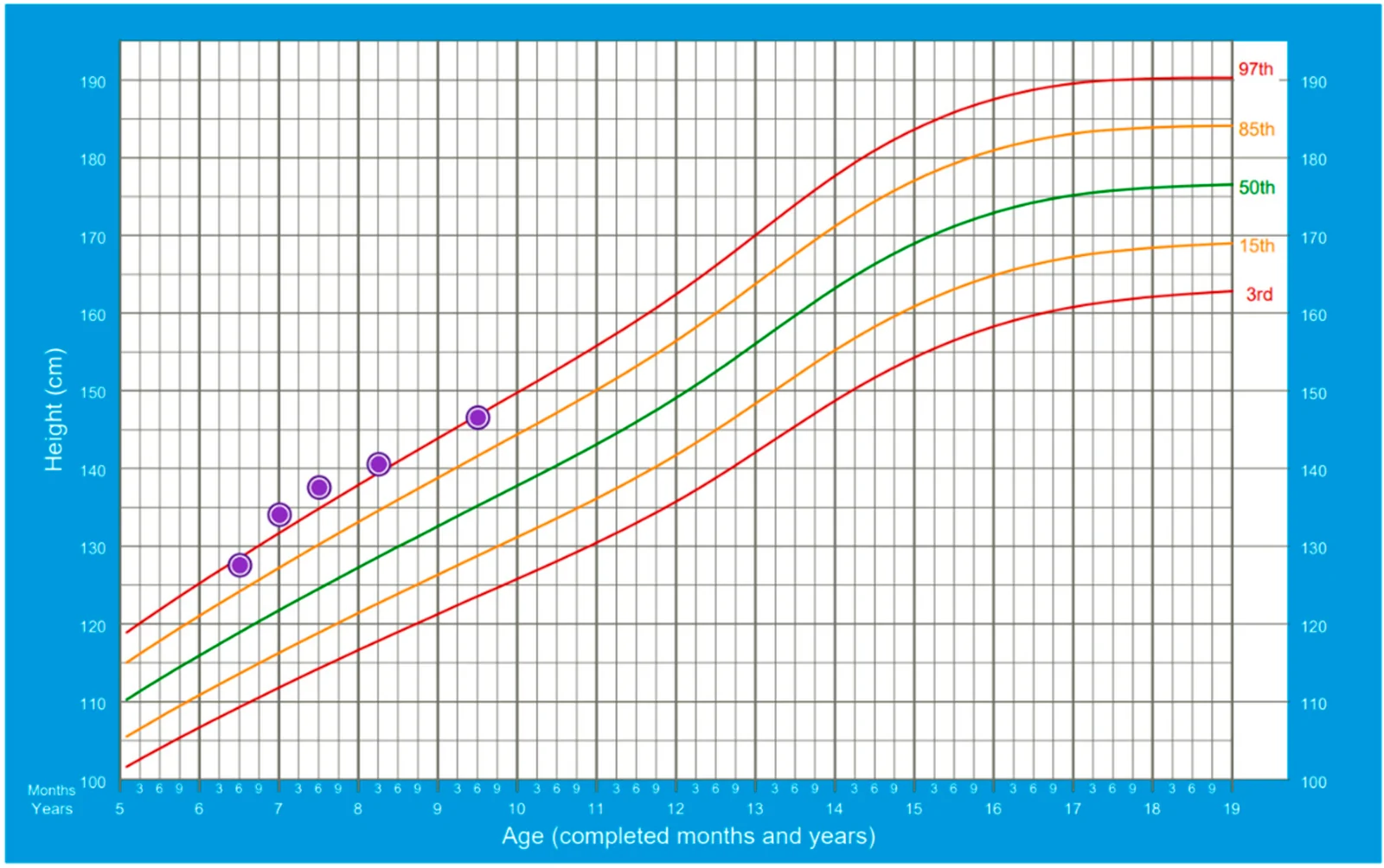
Growth trajectory of the patient plotted against CDC growth reference percentiles for boys aged 5–19 years. Serial anthropometric measurements are shown as purple dots.

**Figure 4:**
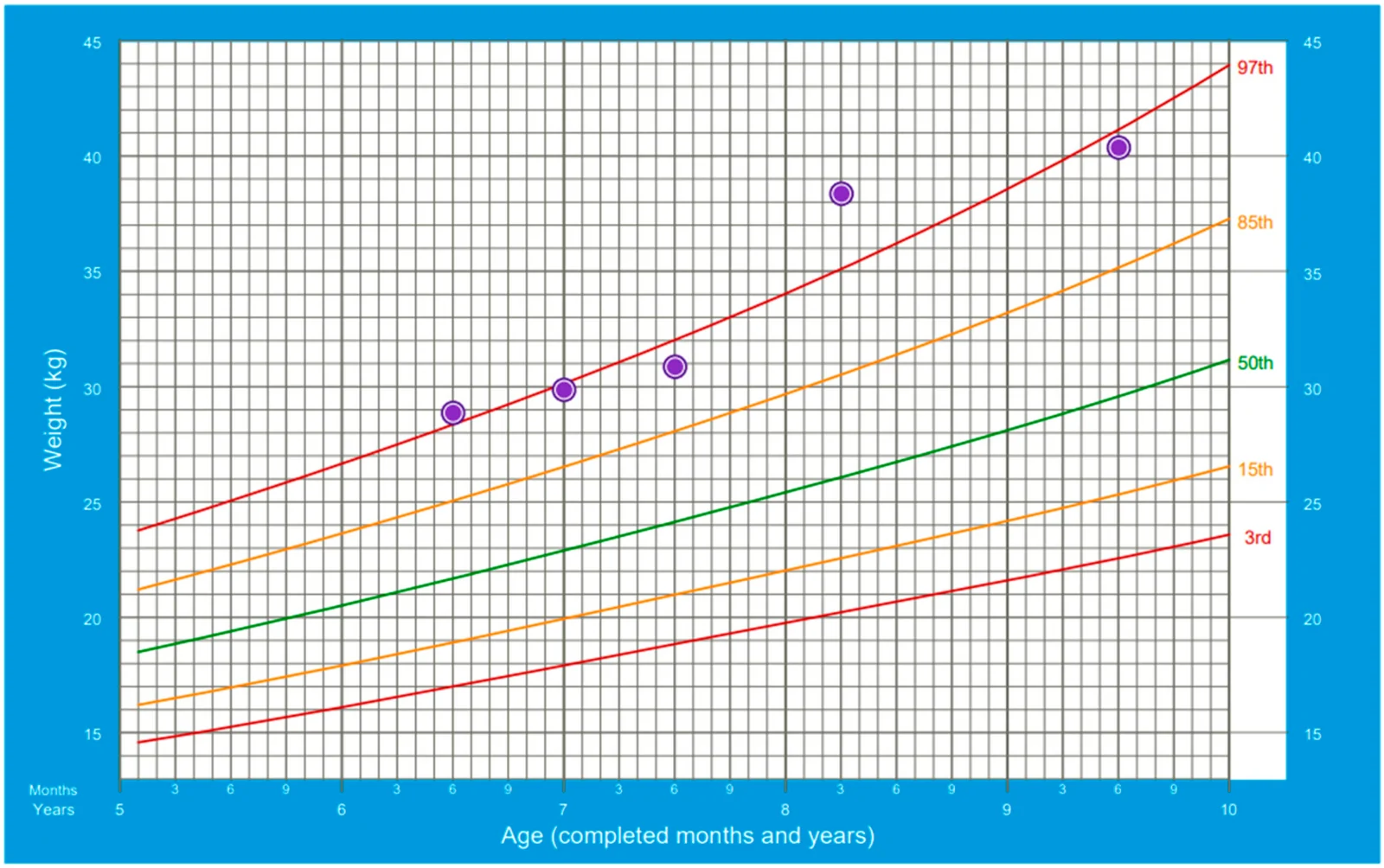
Weight trajectory of the patient plotted against CDC growth reference percentiles for boys aged 5–10 years. Serial anthropometric measurements are shown as purple dots.

Following treatment initiation, growth velocity improved from 7.97 cm/year (height SDS +2.98) before treatment to 4.60 cm/year (height SDS +2.00) during treatment with metreleptin. Body weight SDS decreased from +2.33 to +2.04, indicating a reduction in excess weight. Changes in height and weight before and during treatment are illustrated in Table 3 and Figure 3 and Figure 4.

### 3.2. Clinical Case 2

A 5-year-old male patient was referred for evaluation. He was born full-term following the first pregnancy and first delivery to a healthy, non-consanguineous couple also in the western region of Kazakhstan. The pregnancy was complicated by maternal anemia. The patient’s birth weight was 2.600 g and his length was 51 cm, and at birth, his condition was notable for intrauterine fetal hypoxia. At the age of 1 year, the child was hospitalized for bacterial pneumonia.

At 1.3 years of age, family members started noticing a reduction in the patient’s facial fat. Frequent nose and gingival bleeding were also reported. He then developed knee joint pain, multiple episodes of acute respiratory viral infections, recurrent lymphadenitis of unclear etiology, and acute otitis media.

From the age of 1.5 years, abdominal distension, generalized weakness, nausea, vomiting, and abdominal pain became apparent. He was repeatedly evaluated by a gastroenterologist and received treatment with ursodeoxycholic acid. An umbilical hernia was diagnosed at 1.9 years of age. When he was 6 years old, a systemic disease was suspected by the pediatrician due to additional complaints of marked weakness, knee joint pain, and weight loss.

Developmental delay with delayed speech acquisition was evident, and level 2 general speech underdevelopment was diagnosed at 4 years of age. The combination of clinical manifestations raised suspicion of an underlying systemic disease.

**Figure 5:**
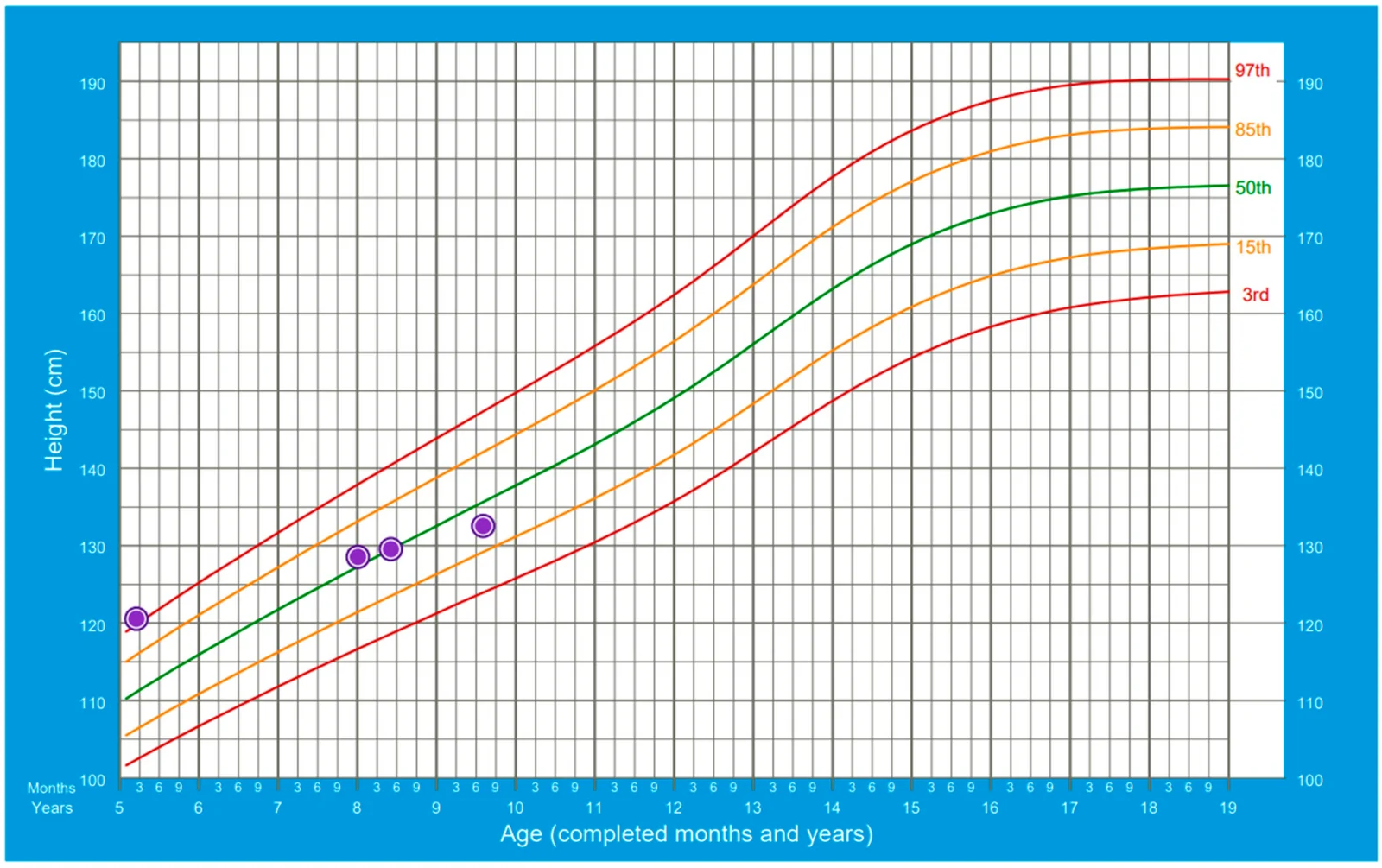
Growth trajectory of the patient plotted against WHO growth reference percentiles for boys aged 5–19 years. Serial anthropometric measurements are shown as purple dots.

**Figure 6:**
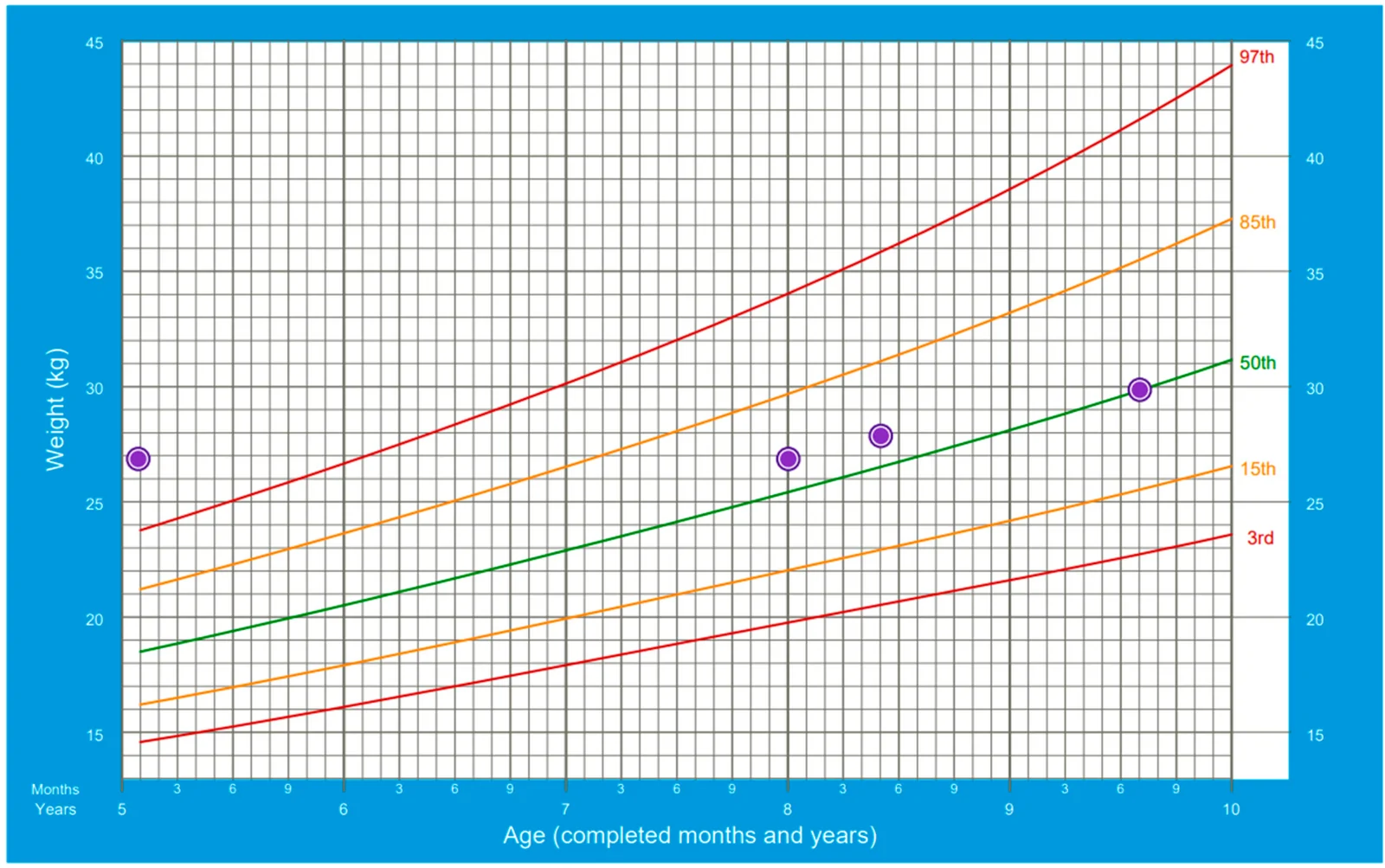
Weight trajectory of the patient plotted against WHO growth reference percentiles for boys aged 5–10 years. Serial anthropometric measurements are shown as purple dots.

On physical examination, prominent skeletal musculature and generalized absence of subcutaneous adipose tissue (involving the face, trunk, and upper and lower extremities) were revealed. Acanthosis nigricans in the neck, axilla, and antecubital fossa were noticed. The abdomen was enlarged and tense on palpation, and the liver was also enlarged on palpation. Changes in height and weight are illustrated in Table 4 and Figure 5 and Figure 6.

Laboratory investigations demonstrated a normal fasting glucose level (4.35 mmol/L), elevated insulin level (84.35 mU/mL), and a glycated hemoglobin (HbA1c) level slightly below normal (4.40%). CBC was significant for severe thrombocytopenia, with a platelet count of 33 × 10^9^/L. The lipid profile showed a low high-density lipoprotein (HDL) level (0.66 mmol/L) and normal low-density lipoprotein (LDL) (1.68 mmol/L) and triglyceride levels (2.15 mmol/L). The liver profile revealed marked elevation in the levels of alanine aminotransferase (ALT) (512.60 U/L) and aspartate aminotransferase (AST) (250.50 U/L).

#### 3.2.1. Molecular Genetic Analysis

Genomic DNA was extracted and analyzed using NGS sequencing (exome sequencing, Illumina); the following genes were analyzed: *AGPAT2*, *BSCL2*, *CAV1*, *CAVIN1*, *CIDEC*, *LIPE*, *LMNA*, *PIK3R1*, *PLIN1*, *POLD1*, *PPARG*, *PSMB8*, *ZMPSTE24*, *FBN1*, *GALC*, *INSR*, *LMNB2*, *MTX2*, *PCNT*, *PCYT1A*, *POLR3A*, and *WRN*. No pathogenic mutations were detected; therefore, various types of lipodystrophies were not confirmed. 

#### 3.2.2. Diagnostic Workup

The patient had normal fat distribution at birth followed by its gradual loss around 1.3 years of age. Accordingly, this makes CGL substantially less likely. Genetic testing did not determine any pathogenic variants associated with lipodystrophy; however, this negative result was supportive rather than diagnostic of an acquired etiology. The later onset of progressive generalized fat loss after an initially normal adipose tissue distribution therefore represented the principal clinical feature supporting AGL.

Given the thrombocytopenia, hepatosplenomegaly, and other systemic findings, an extended diagnostic evaluation was performed to investigate infectious, hematological, and structural disorders that could mimic or coexist with AGL. Testing for hepatitis B, hepatitis C, and HIV was negative, thereby excluding infectious causes of secondary thrombocytopenia; bone marrow examination showed megakaryocytic hyperplasia with reduced platelet budding and no excess blasts; and flow-cytometric immunophenotyping found no evidence of acute leukemia. Based on clinical and hematological findings, chronic immune thrombocytopenia was diagnosed. Imaging demonstrated hepatosplenomegaly; nephromegaly; diffuse changes in the liver, pancreas, and kidneys; cardiomegaly; and left ventricular hypertrophy, although cardiac function was preserved.

The concomitant autoimmune thrombocytopenia provided additional support for the diagnosis. No episode of panniculitis was documented in the available medical records. Complement levels and autoantibodies were not investigated; therefore, a complement-associated or specific autoimmune mechanism could not be assessed.

Overall, the patient was diagnosed with AGL based on acquired, progressive, generalized loss of adipose tissue after an initially normal fat distribution, associated metabolic abnormalities and the absence of an identified genetic cause of inherited lipodystrophy.

#### 3.2.3. Management and Outcome Characteristics

A hypocaloric, low-fat, low-carbohydrate diet with a total caloric intake of approximately 1300 kcal/day was recommended. The diet limited fried and spicy foods, excluded high-glycemic carbohydrates, and emphasized boiled, steamed, or baked dishes. However, as the patient received metreleptin therapy for a short period, hyperphagia persisted, making it difficult for the patient to adhere to the diet. 

Ursodeoxycholic acid (250 mg per os once a day) was prescribed to manage MASLD. Metreleptin therapy at a dosage of 0.06 mg/kg/day (1.32 mg) was initiated after the diagnosis was made.

One month after starting metreleptin therapy, the patient was admitted to the pediatric intensive care unit due to bleeding episodes becoming more frequent in the setting of persistent thrombocytopenia and insufficient response to hematological treatment. Given this adverse event, metreleptin therapy was temporarily discontinued with gradual dose reduction. The temporal association between metreleptin therapy and the bleeding episode was acknowledged; however, causality could not be established because thrombocytopenia was present before treatment initiation.

Metreleptin had been prescribed according to approved indications, as it is recommended for the treatment of generalized lipodystrophy and can be administered in pediatric patients aged 2 years and older.

Following reassessment by hematologists, treatment with eltrombopag (Revolade) 50 mg/day was initiated, resulting in improvement in thrombocytopenia. The patient remains under hematological follow-up.

Subsequent inpatient assessment found secondary arterial hypertension, for which antihypertensive therapy with enalapril 0.1 mg/kg/day was started.

The patient continues multidisciplinary follow-up for monitoring of metabolic and systemic complications associated with acquired generalized lipodystrophy.

## 4. Discussion

We present cases of CGL and AGL to demonstrate differences in clinical courses and management approaches. In the first clinical case, the patient was diagnosed with CGL type 2, attributed to a homozygous variant, c.823C>T (p.R275*), in exon 8 of the *BSCL2* gene. The *BSCL2*-encoded protein, seipin, plays a crucial role in lipid droplet formation and adipocyte differentiation, and its dysfunction results in severe lipodystrophy and metabolic abnormalities [23,24].

Previous studies have described a variety of pathogenic *BSCL2* variants in patients with CGL2. Liu et al. reported four patients with *BSCL2*-associated CGL who presented with generalized lipoatrophy, skeletal muscular hypertrophy, hepatomegaly, hypertriglyceridemia, hyperinsulinemia, and hepatic dysfunction in the infantile period [25]. Further reports have shown significant genetic and phenotypic variability among patients with CGL2 [26,27,28]. These findings are consistent with the early and severe metabolic features found in our first patient and provide broader context for the identified homozygous *BSCL2* variant.

The abnormal development of adipose tissue and excessive lipid fluxes trigger the liver to produce triglycerides and very low-density lipoproteins, and hinder muscular glucose uptake [29]. Leptin deficiency aggravates hunger, which also worsens the metabolic profile, leading to severe clinical complications [29]. Patients suffer from hyperglycemia leading to diabetes and hypertriglyceridemia—which can be complicated; acute pancreatitis, cardiovascular problems, secondary nephropathy and nonalcoholic fatty liver diseases, skeletal muscle hypertrophy, and neurological involvement (delayed speech development) were all noticed in the first case [3]. Cardiac abnormalities inherent to CGL type 2 involve ventricular dysfunction and hypertrophic cardiomyopathy [30]; the onset of the latter is correlated with increased plasma insulin levels that stimulate the type 1 insulin-like growth factor (IGF-1) receptors in myocardial tissue [31].

Importantly, the *BSCL2* c.823C>T (p. Arg275Ter; p.R275*) variant is an ultra-rare pathogenic nonsense variant. It is present at an extremely low allele frequency in population databases and has been previously reported in patients with congenital generalized lipodystrophy type 2, including homozygous cases, but remains exceptionally rare worldwide [32,33]. The identification of a homozygous pathogenic *BSCL2* variant is particularly relevant in the context of Central Asia: consanguineous marriages have been reported in several populations of West, Central, and South Asia, and consanguinity increases the probability of homozygosity for rare pathogenic alleles, thereby elevating the risk of autosomal recessive disorders [34]. Because CGL type 2 is an autosomal recessive disorder caused by pathogenic variants in *BSCL2*, affected patients from populations with endogamy, geographic isolation, or consanguinity may provide important information on the regional mutation profile. Although founder or recurrent *BSCL2* variants have been reported in other populations, to our knowledge, no well-characterized Central Asian founder variant in *BSCL2* has yet been formally established [35,36,37]. Therefore, we highlight the need for systematic genetic studies in endogamic or consanguineous populations in Central Asia.

Next, differentiating AGL from CGL is clinically relevant since both lipodystrophies have the same phenotype but differ in the disease onset, genetic component, pathogenesis, and genetic counseling options. CGL usually develops from birth or early infancy, and the underlying pathogenesis is inherited pathogenic variants in genes responsible for the development and homeostasis of adipose tissue. Conversely, in AGL, patients are born with normal adipose tissue distribution and develop adipose tissue loss later during childhood or adolescence. The exact etiology is not completely understood and is usually linked to immune-mediated or inflammatory destruction of fat cells. The most common autoimmune conditions associated with AGL are Sjögren syndrome, juvenile dermatomyositis, Hashimoto’s thyroiditis, autoimmune hepatitis, juvenile idiopathic arthritis, and autoimmune cytopenias [3,12]. Inflammatory causes of AGL are linked to panniculitis; inflammatory subcutaneous nodules or episodes of panniculitis may result in localized fat tissue atrophy, which may progress to generalized lipodystrophy over time [3,15]. The remaining cases of AGL represent idiopathic forms with no clear trigger, autoimmune condition, or panniculitis detected.

In the second case, the absence of early-infantile presentation, negative multigene panel testing, and the presence of autoimmune thrombocytopenia favored AGL over CGL. A comparable case of AGL concomitant with autoimmune thrombocytopenia has previously been reported in an adult patient [38]. Although the age at presentation differed from that of our case, the coexistence of generalized lipodystrophy and immune-mediated thrombocytopenia in both cases depicts the association of autoimmune processes and AGL. The present case extends the available literature by documenting a similar presentation in a pediatric patient and adds to the limited evidence on immune-mediated forms of lipodystrophy in Central Asia.

Recent clinical guidance highlights that lipodystrophy diagnosis should incorporate the pattern and progression of fat-tissue loss with metabolic assessment and targeted genetic or immunological testing, followed by multidisciplinary management and follow-up [2,39]. Moreover, the wide phenotypic variety of *BSCL2*-associated disorders, including progressive encephalopathy with or without lipodystrophy, demonstrate the need for careful neurological monitoring in affected children [40]. Our cases emphasize the role of this coordinated approach while also showing the need for expanded access to molecular genetic testing, targeted therapies, and expert multidisciplinary care.

Furthermore, metreleptin stands out as the only specialized treatment against the metabolic complications of lipodystrophy [15,16,17]. Along with its direct physiological effects, metreleptin has a psychosocial impact on patients’ lives. Children may experience bullying due to their masculine appearance and fat loss, which can lead to depression and social isolation. After treatment, behavioral changes were noted in the first case after the reduction in hyperphagia and irritability, allowing for better concentration and learning, despite speech impairments. Improvements in metabolic health simplified the treatment regimen and decreased the burden of polypharmacy. Enhanced patient adherence may also be associated with the use of a single drug, such as metreleptin, thereby increasing treatment compliance and efficacy.

Nutritional therapy is an important component of the multidisciplinary treatment of generalized lipodystrophy and should be individualized according to age, growth, and metabolic status, with particular attention paid to the control of hypertriglyceridemia and hyperglycemia. In children, dietary management should involve a dietitian to ensure adequate energy intake and appropriate dietary composition while considering growth and developmental needs. In our patients, specialized dietary counseling was not provided due to the absence of a dietitian at the clinic, and whole-body DXA was not performed because the patients’ age and body weight did not meet the requirements for the examination, which represents a limitation. Future studies may benefit from ultrasound stratigraphy of subcutaneous adipose and muscle tissue to provide additional information on changes in body composition during nutritional interventions.

Given their rarity and heterogeneity, lipodystrophy disorders frequently remain overlooked or misdiagnosed [3]. Emphasizing the value of a systematic assessment is vital in managing rare diseases like CGL and AGL. A multidisciplinary team of endocrinologists, geneticists, pediatricians, cardiologists, dietitians, and therapists provides a holistic approach to patient care, addressing metabolic, physical, and psychosocial aspects of the disease. Raising awareness among general practitioners (GPs) and pediatric specialists about early screening, clinical manifestation, and diagnostic criteria is critical for on-time diagnoses and interventions.

Based on a literature search, the available data on lipodystrophy in Central Asia is extremely scarce: we identified no epidemiological studies or case reports, case series, or observational studies from the region. The absence of published cases should not be interpreted as evidence of a low disease prevalence, as under-recognition and misdiagnosis may represent contributing factors. Considering this, our literature review and case series provide valuable regional evidence and address a significant gap in existing literature.

This study has several limitations. First, the regional literature search was targeted rather than systematic. Furthermore, the second case was incompletely characterized in some aspects; in particular, complement levels and autoantibody testing were not available, limiting full assessment of possible immune-mediated mechanisms. Standardized clinical images and objective body-composition assessments, such as DXA or MRI, were also not obtained during routine clinical care because of limited availability and practical constraints related to the patients’ young age. In addition, the follow-up duration differed between the two patients. Finally, this two-patient case series from a single-centre setting cannot establish the prevalence or degree of underdiagnosis of lipodystrophy in Central Asia; the scarcity of published cases should therefore be interpreted as limited regional representation in the literature rather than direct evidence of underdiagnosis. Relevant cases of lipodystrophy may be reported in local languages or remain unpublished, thereby limiting the completeness of the review. Because the condition is extremely rare and follow-up is limited, long-term outcomes of the disease remain difficult to assess.

### 4.1. Challenges and Future Directions for Lipodystrophy Care in Central Asia

#### 4.1.1. Low Disease Awareness

Lipodystrophy remains poorly recognized among healthcare professionals in Central Asia. Because of its rarity and heterogeneous phenotype, patients are frequently misdiagnosed as having atypical diabetes, severe insulin resistance syndrome, malnutrition, or atypical obesity phenotypes; consequently, referrals to specialized endocrinology services may be substantially delayed.

We propose increasing healthcare professionals’ awareness of rare disorders through medical education programs at the institutional level. Educational initiatives targeting endocrinologists, pediatricians, hepatologists, and GPs may facilitate earlier recognition of characteristic clinical features, including generalized fat loss, muscular appearance, acanthosis nigricans, hypertriglyceridemia, and early-onset diabetes mellitus.

#### 4.1.2. Limited Access to Genetic Testing

Genetic confirmation remains an essential component of the diagnostic workup, particularly for congenital forms of lipodystrophy. However, access to molecular diagnostics in Central Asia is often limited by financial constraints, lack of local laboratory infrastructure, and dependence on international referral laboratories.

Expanding access to affordable genetic testing through international partnerships and regional laboratories could significantly optimize diagnostic approach, facilitate family screening, and ultimately enhance patients’ quality of life and improve long-term survival.

#### 4.1.3. Absence of a Regional Rare-Disease Registry

Currently, there is no population-based disease registry in Central Asia, resulting in unknown disease burden in the region, delayed diagnosis, and limited awareness of genotypes that could be specific to Central Asia.

Developing a regional lipodystrophy registry and multidisciplinary rare-disease centers may help estimate disease burden and facilitate collaborative research.

#### 4.1.4. Limited Access to Advanced Therapies

Access to disease-specific therapies, including metreleptin, remains challenging in many countries due to cost, regulatory barriers, and limited availability. As a result, treatment often relies on conventional approaches focusing on controlling diabetes, hypertriglyceridemia, and hepatic complications.

Improving access to specialized therapies may substantially enhance quality of life and long-term outcomes for affected individuals.

## 5. Conclusions

The published evidence on lipodystrophy in Central Asia is extremely limited. Our case series highlights possible barriers to diagnosis and management, including low disease awareness, limited access to genetic testing, fragmented multidisciplinary care, and restricted availability of specialized therapies. These factors may contribute to the underreporting of lipodystrophy in the region.

Furthermore, there is a need for regional collaboration and infrastructure development. Establishing referral systems, improving physician education, and expanding access to specialized diagnostic and therapeutic resources may facilitate earlier diagnosis and improve outcomes for patients with lipodystrophy across Central Asia.

Beyond presenting individual cases, this study serves as a call to action to increase recognition of lipodystrophy as an important, rare disease within the region and to stimulate future collaborative research efforts.

## Figures and Tables

**Table 1:** Metabolic changes before and during metreleptin therapy.

Findings	Before Treatment	6 Months After	12 Months After	21 Months After	36 Months After	Reference Range
Plasma glucose (mmol/L)	19.04	5.99	5.11	4.48	5.45	(3.30–5.60)
HbA1c (%)	7.76%	5.15	5.14	5.23	5.66	(4.00–6.00)
Plasma C-peptide (ng/mL)	8.01	3.20	2.59	2.35	2.95	(1.10–4.40)
Insulin (mcME/mL)	92.16	15.02	13.62	15.50	20.58	(2.60–24.90)

**Table 2:** Lipid profile changes before and during metreleptin therapy.

Findings	Before Treatment	6 Months After	12 Months After	21 Months After	36 Months After	Reference Range
Total cholesterol (mmol/L)	4.52	3.92	3.84	3.70	3.04	(0.00–5.20)
LDL cholesterol (mmol/L)	1.84	2.85	2.75	2.37	1.99	(1.63–3.34)
HDL cholesterol (mmol/L)	0.59	0.93	1.01	0.92	0.76	(0.98–1.94)
Triglyceride (mmol/L)	9.71	1.90	1.14	1.93	1.57	(0.00–1.70)

**Table 3:** Anthropometric measurements before and during treatment.

	Age	2 Years 7 Months	4 Years 5 Months	6 Years 6 Months (Treatment Initiation)	7 Years	7 Years 6 Months	8 Years 3 Months	9 Years 6 Months
Attribute								
Height (cm)		101	118	128	134.5	138	141	147
SDS Height		+1.9	+2.63	+1.83	+2.47	+2.51	+2.2	+1.94
Weight (kg)		17	25	29	30	31	38.5	40.5
SDS Weight		+1.9	+2.76	+2.03	+1.88	+1.71	+2.43	+1.83

**Table 4:** Anthropometric measurements during follow-up.

	Age	4 Years 11 Months	8 Years 0 Months	8 Years 5 Months	9 Years 7 Months
Attribute					
Height (cm)		121	129	130	133
SDS Height		+2.39	+0.29	+0.03	−0.42
Weight (kg)		27	27	28	30
SDS Weight		+1.33	+0.40	+0.32	+0.03

## Data Availability

The original contributions presented in this study are included in the article. Further inquiries can be directed to the corresponding author.

## Abbreviations

The following abbreviations are used in this manuscript:

ACMG/AMP	American College of Medical Genetics and Genomics and the Association for Molecular Pathology
AGL	Acquired generalized lipodystrophy
AGPAT2	1-acylglycerol-3-phosphate O-acyltransferase 2
APL	Acquired partial lipodystrophy
ALT	Alanine aminotransferase
AST	Aspartate aminotransferase
BMI	Body mass index
BP	Blood pressure
BSCL	Berardinelli–Seip congenital lipodystrophy
CAV1	Caveolin
CAVIN1	Caveolae-associated protein 1
CBC	Complete blood count
CDC	Centers for Disease Control and Prevention
CGL	Congenital generalized lipodystrophy
CIDEC	Cell death-inducing DFFA-like effector C
DNA	Deoxyribonucleic acid
EF	Ejection fraction
EMA	European Medicines Agency
FBN1	Fibrillin 1
FPL	Familial partial lipodystrophy
FPG	Fasting plasma glucose
GL	Generalized lipodystrophy
GP	General practitioner
GALC	Galactosylceramidase
HbA1c	Glycated hemoglobin
HDL	High-density lipoprotein
IFG	Impaired fasting glucose
IGF-1	Insulin-like growth factor
INSR	Insulin receptor
LDL	Low-density lipoprotein
LIPE	Lipase E, hormone sensitive type
LMNA	Lamin A/C
LMNB2	Lamin B2
MTX2	Metaxin 2
MASLD	Metabolic dysfunction-associated steatotic liver disease
NGS	Next-generation sequencing
OMIM	Online Mendelian Inheritance in Man
PMOS	Polyendocrine metabolic ovarian syndrome
PTRF	Polymerase I and transcript release factor
PIK3R1	Phosphoinositide-3-Kinase Regulatory Subunit 1
PLIN1	Perilipin 1
POLD1	DNA polymerase delta 1, catalytic subunit
PPARG	Peroxisome proliferator-activated receptor gamma
PSMB8	Proteasome 20S Subunit Beta 8
PCNT	Pericentrin
PCYT1A	Phosphate cytidylyltransferase 1, choline, alpha
POLR3A	RNA polymerase III subunit A
SDS	Standard deviation score
sPAP	Systolic pulmonary arterial pressure
UMC	University Medical Center
USA	United States of America
WES	Whole-exome sequencing
WRN	Werner syndrome helicase
ZMPSTE24	Zinc metallopeptidase STE24
